# Unveiling Clinical Phenotypes in Chronic Chikungunya Disease: Insights from a Brazilian Observational Study

**DOI:** 10.3390/tropicalmed11050140

**Published:** 2026-05-19

**Authors:** Karen Santos Lima, Adriane Paz Rocha, Alice Lanna Damásio Castro, Anna Carolina Faria Moreira Gomes Tavares, Flávia Patrícia Sena Teixeira Santos, Gilda Aparecida Ferrreira, Livia Barbara Cordeiro Alves, Josiane Lino dos Santos Frattari, Juliana Froeseler Fittipaldi, Lucas Borba Paulino Coelho, Maria Fernanda Brandão de Resende Guimarães, Pedro Ribeiro de Jesus Almeida, Último Libânio Costa, Cristina Costa Duarte Lanna, Gustavo Gomes Resende, Mauro Martins Teixeira

**Affiliations:** 1 Hospital das Clínicas, Universidade Federal de Minas Gerais, Av. Prof. Alfredo Balena, 110, Santa Efigênia, Belo Horizonte 30130-100, MG, Brazil; karenslima@yahoo.com.br (K.S.L.); adrianepazrocha@gmail.com (A.P.R.); annacgtavares@gmail.com (A.C.F.M.G.T.); flaviapatriciasena@gmail.com (F.P.S.T.S.); mfbresende@yahoo.com.br (M.F.B.d.R.G.); gustavo.resende@ebserh.gov.br (G.G.R.); 2 Unidade de Pesquisa Clínica, Centro de Terapias Avançadas e Inovadoras, Universidade Federal de Minas Gerais, Rua Carlos Pinheiro Chagas, 20, Jardim Montanhês, Belo Horizonte 30750-150, MG, Brazil; alannadamasio@gmail.com (A.L.D.C.); alvesliviab@gmail.com (L.B.C.A.); joseanelinodossantosfrattari@gmail.com (J.L.d.S.F.); jubiologia@gmail.com (J.F.F.); lucasborbapc@gmail.com (L.B.P.C.); ultimolibanio@gmail.com (Ú.L.C.); mmtex.ufmg@gmail.com (M.M.T.); 3 Departamento do Aparelho Locomotor, Faculdade de Medicina, Universidade Federal de Minas Gerais, Av. Prof. Alfredo Balena, 190, Santa Efigênia, Belo Horizonte 30130-100, MG, Brazil; gildaferreira9@gmail.com (G.A.F.); duartelanna@gmail.com (C.C.D.L.)

**Keywords:** chikungunya fever, phenotypes, polyarthritis, oligoarthritis, axial

## Abstract

Chronic chikungunya disease (CCD) affects approximately 30–50% of infected individuals and is associated with persistent inflammatory arthritis, chronic pain, and long-term functional disability. We conducted a prospective observational cohort study including 584 patients with laboratory-confirmed chikungunya infection, evaluated between 3 and 12 months after acute infection, to better understand the natural history, risk factors, clinical presentation, and treatment patterns of CCD. Here, we present a cross-sectional analysis derived from this cohort. Older age, female sex, and higher body mass index were identified as major risk factors for CCD. Four distinct clinical phenotypes were identified: Axial (12.2%), defined by inflammatory axial pain regardless of peripheral manifestations; Oligoarthritis (18.1%), defined by fewer than four swollen joints; Polyarthritis (10.6%), defined by four or more swollen joints; and Pain without Swelling (70.4%), characterized by myalgia and/or arthralgia in the absence of objective inflammatory findings on physical examination. The axial phenotype could overlap with peripheral phenotypes, whereas oligoarthritis, polyarthritis, and pain without swelling were mutually exclusive categories. These phenotypes differed substantially in symptom burden and clinical impact. Patients with the Pain without Swelling phenotype had longer symptom duration, whereas Axial and Polyarthritis phenotypes were associated with greater functional impairment and higher disease burden. These findings reinforce the clinical heterogeneity of CCD and support the potential value of phenotype-based approaches for clinical management and future therapeutic research.

## 1. Introduction

Chikungunya virus (CHIKV) is an arthropod-borne alphavirus transmitted mainly by *Aedes aegypti* and *Aedes albopictus*, historically endemic in Africa and Asia and is characterized by marked seasonality in tropical regions that favor the vector proliferation [1,2,3]. Over recent decades, the global burden of chikungunya fever (CHIKF) has increased substantially, driven by the geographic expansion of mosquito vectors, climate and environmental changes, urbanization, and human mobility. Although vector control remains the cornerstone of prevention, it has proven insufficient to curb the spread of the disease [1,4,5].

Until 2013, CHIKF in the Americas was limited to imported cases; however, the introduction of the virus in the Caribbean marked the beginning of sustained local transmission and rapid continental dissemination. In Brazil, autochthonous transmission was first documented in 2014, followed by successive epidemic waves [6,7]. While approximately 1.7 million CHIKF cases were officially reported in Brazil between 2014 and 2024, seroprevalence-based modeling studies suggest that the true cumulative number of infections during the same period may have reached up to 40 million individuals, equivalent to nearly 18% of the population [8,9]. As of 2026, chikungunya is firmly established as an endemic–epidemic disease in the Americas, with recurrent outbreaks and a substantial cumulative public health impact, particularly due to its chronic musculoskeletal and neuropathic manifestations [10].

Clinically, CHIKV infection typically presents as an acute febrile illness characterized by fever, polyarthralgia, myalgia, headache, rash, nausea, and fatigue [3,5,11,12]. Although many patients recover within weeks, a considerable proportion progress to a chronic phase, with persistent symptoms lasting beyond three months. Chronic chikungunya disease (CCD) affects approximately 30–50% of infected individuals, with significant regional variability. It is associated with persistent inflammatory arthritis and/or arthralgia, stiffness, fatigue, sleep disturbances, mood disorders, neuropathic and nociplastic pain, and long-term functional disability [10,13,14,15,16]. These manifestations result in a marked impairment of quality of life and represent one of the most disabling outcomes among arboviral diseases [11,17,18,19].

The disease course is classically divided into acute (≤3 weeks), subacute (3 weeks to 3 months), and chronic (>3 months) phases, with symptoms varying in type and intensity across individuals [2,5,12]. In the chronic phase, patients may present with heterogeneous rheumatological phenotypes, including inflammatory arthritis resembling rheumatoid arthritis or spondyloarthritis, enthesitis, soft tissue rheumatism, and mixed inflammatory and non-inflammatory pain syndromes [4,10,20,21]. Long-term follow-up studies have demonstrated persistence of symptoms for several years after infection, and in some cases, irreversible musculoskeletal damage [4,19,22,23].

The mechanisms underlying CCD remain incompletely understood and likely involve multiple immunopathological pathways. Proposed mechanisms include viral persistence in synovial macrophages, sustained Th1/Th17 inflammatory responses, inadequate resolution of inflammation, and epigenetic modifications. Elevated levels of proinflammatory cytokines, including tumor necrosis factor (TNF), interleukin-1β, interleukin-6, and interleukin-17, have been associated with chronic inflammatory manifestations in CCD. However, the relative contribution of these pathways to disease persistence and phenotype expression remains incompletely understood [12,24,25,26,27]. In this context, CHIKV infection has also been proposed as a potential trigger for autoimmune conditions in genetically susceptible individuals [4,23,25].

In Brazil, the estimated prevalence of CCD varies widely across regions, ranging from approximately 35% to over 70%, with older age, female sex, and higher inflammatory burden during the acute phase consistently identified as major risk factors for chronicity [10,13,14,15,16,28]. Despite the high burden of chronic disease, clinical heterogeneity remains insufficiently characterized, limiting the development of personalized management strategies.

Therefore, this study aimed to evaluate the clinical and demographic characteristics of CCD in a Brazilian cohort and to identify distinct phenotypic clusters. By characterizing these clusters, we seek to improve the understanding of disease heterogeneity and to provide a framework for more targeted therapeutic approaches and optimized long-term patient care.

## 2. Materials and Methods

### 2.1. Study Design and Participants

This was a cross-sectional analysis derived from a prospective observational cohort conducted in the city of Contagem, Minas Gerais State, Brazil. The study protocol was approved by the Research Ethics Committee of the Universidade Federal de Minas Gerais (CAAE: 71268823.8.0000.5149). Individuals of any age with laboratory-confirmed CHIKV infection (IgM serology or RT-PCR), identified through the local epidemiological surveillance registry and with a valid phone number, were invited to participate. Written informed consent was obtained from all participants or legal guardians.

### 2.2. Clinical Assessment and Grouping

Participants were evaluated between three and 12 months after symptom onset and categorized into two groups: (1) asymptomatic individuals in whom typical chikungunya-related symptoms were no longer present (Control group), and (2) individuals with persistent musculoskeletal symptoms (CCD group). All participants underwent an initial clinical and rheumatologic assessment (V1) and completed questionnaires assessing pain, functionality, health-related quality of life, and mental health. Visit 1 was followed by a scheduled follow-up visit 12 months later (V2). Additional visits were performed between V1 and V2 when clinically indicated. Before study initiation, the study team underwent alignment meetings to standardize clinical assessments and harmonize definitions of inflammatory axial pain and tender/swollen joint counts across investigators. Phenotype classification was performed by trained rheumatologists based on standardized clinical assessment at V1.

### 2.3. Data Collection

Data collected included demographic and clinical characteristics, and a comprehensive rheumatologic assessment comprising medical history and physical examination. Pain intensity, fatigue, and perceived disease activity, as reported by both patients and physicians, were assessed using a 10 cm Visual Analog Scale (VAS). Tender joint count (TJC-68), swollen joint count (SJC-66), and enthesitis count based on sites included in SPARCC (Spondyloarthritis Research Consortium of Canada) [29] and MASES (Maastricht Ankylosing Spondylitis Enthesitis Score) [30] scores were recorded. The presence of axial inflammatory pain and carpal tunnel syndrome (CTS) was determined clinically.

Axial inflammatory pain was defined by pain occurring predominantly in the morning and at night, improving with physical activity and aggravated by rest, affecting any region of the vertebral spine or the buttocks (sacroiliac joints). Clinical carpal tunnel syndrome (CTS) was defined by the presence of numbness, tingling, or pain in the hand, wrist, or distal forearm, predominantly affecting the thumb, index, and middle fingers, in association with a positive Phalen, Tinel, or Durkan test.

Laboratory assessment included measurement of C-reactive protein (CRP) levels.

Patient-reported outcomes included the Health Assessment Questionnaire (HAQ) [31], Patient Health Questionnaire-9 (PHQ-9) [32], 12-Item Short-Form Health Survey (SF-12, including physical and mental component summaries) [33], and Generalized Anxiety Disorder-7 (GAD-7) [34]. Neuropathic pain was assessed using the Douleur Neuropathique en 4 questions (DN4) questionnaire [35]. All instruments, previously translated and validated for Brazilian Portuguese, were administered by a trained nurse. Interpretation guidelines for each questionnaire are detailed in Table 1. The study adhered to the STROBE (STrengthening the Reporting of OBservational studies in Epidemiology) reporting guidelines. STROBE checklist is available as Supplementary Material [36].

### 2.4. Statistical Analysis

Descriptive statistics were used to summarize demographic and clinical characteristics. Categorical variables were compared using Pearson’s chi-square test or Fisher’s exact test, as appropriate, and continuous variables were compared using Student’s *t*-test or the Wilcoxon rank-sum test, according to data distribution.

Binary logistic regression models were applied to estimate associations between clinical and demographic variables and the presence of chronic chikungunya disease (CCD), using the control group as the reference category. To investigate factors associated with specific CCD phenotypes, multinomial logistic regression models were fitted with CCD phenotype as the dependent categorical outcome. The Pain without Swelling phenotype was used as the reference category, and results were reported as odds ratios (ORs) with 95% confidence intervals (95% CIs). Initial models included clinically relevant variables, followed by stepwise refinement to obtain parsimonious final models. Multicollinearity was assessed using variance inflation factors (VIF), and model fit was evaluated through residual and goodness-of-fit analyses. Analyses were performed using complete-case data, with participants presenting missing values excluded from the corresponding models.

Given the exploratory nature of the analyses, no formal correction for multiple comparisons was applied, and findings should be interpreted accordingly. All tests were two-sided, and statistical significance was defined as *p* < 0.05. Statistical analyses were performed using R software, version 4.3.2, with support from the packages car, MASS, pROC, ResourceSelection, hnp, and eulerr.

## 3. Results

A total of 2178 patients with RT-PCR- or IgM-confirmed CHIKV infection were registered by the Epidemiological Surveillance Center. Among them, 1860 had a valid phone number and were contacted. Of these, 686 contact attempts were unsuccessful. Ultimately, 584 patients were included in the study.

Among the 584 participants, 426 (73%) were categorized as the CCD group. Compared to controls (*n* = 158), CCD patients were older [mean (SD): 49.8 (16.4) vs. 44.3 (23.4) years; *p* = 0.001], more frequently female (74% vs. 46%; *p* < 0.0001), had higher body mass index [BMI; mean (SD): 28.9 (6.6) vs. 26.8 (6.6) kg/m^2^; *p* = 0.001], and presented a shorter interval since infection onset [mean (SD): 249.8 (69.6) vs. 268.7 (56.7) days; *p* = 0.001]. The demographic and clinical characteristics of both groups are summarized in Table 2.

Functionality, quality of life, and mental health outcomes were compared between the CCD and control groups, as shown in Table 3. Across all validated questionnaires, the control group demonstrated significantly better scores.

**Figure 1:**
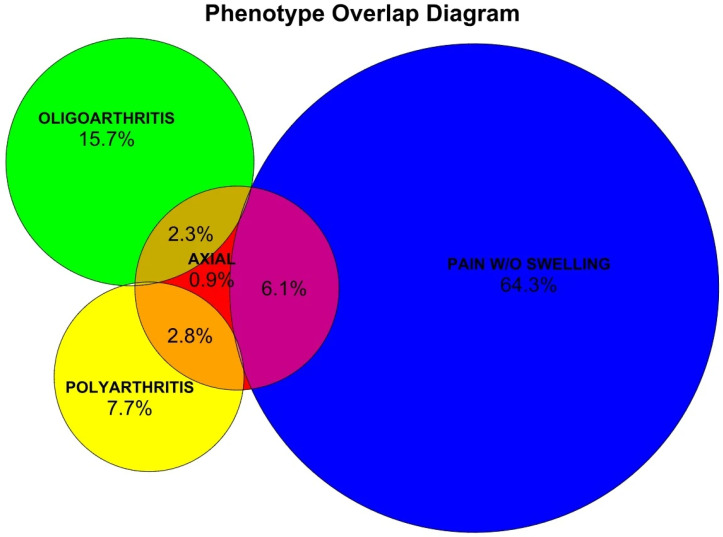
Euler diagram showing the overlap between CCD phenotypes.

The CCD group was further subclassified into four clinical phenotypes, as illustrated in Figure 1, defined as follows: (1) Axial—presence of inflammatory axial pain, regardless of peripheral manifestations; (2) Oligoarthritis—fewer than four swollen joints (SJC-66 < 4); (3) Polyarthritis—four or more swollen joints (SJC-66 ≥ 4); and (4) Pain without swelling—presence of myalgia and/or arthralgia in the absence of objective inflammatory findings on physical examination (i.e., no edema, erythema, or local warmth). Accordingly, the axial phenotype could overlap with any of the peripheral subtypes, whereas oligoarthritis, polyarthritis, and pain without swelling were mutually exclusive categories.

Inflammatory axial pain was identified in 52 participants (12.2%); oligoarthritis in 77 (18.1%); polyarthritis in 45 (10.6%); and 300 participants (70.4%) were classified under the pain without swelling subgroup.

A comparison of the physical examination characteristics and the inflammatory biomarker values of CCD and control groups is summarized in Table 4. Statistically significant differences were observed across most clinical parameters. Notably, levels of C-reactive protein (CRP), the only laboratory marker assessed, did not differ between groups.

Patients were asked about prior treatments used to manage pain since the onset of CHIKV infection. Reported medications included analgesics, non-steroidal anti-inflammatory drugs (NSAIDs), glucocorticoids, opioids, and gabapentinoids. Additionally, disease-modifying agents such as hydroxychloroquine and methotrexate were prescribed in selected cases. Overall, 95% of participants reported the use of at least one pain-related medication. The distribution of medication use across groups is presented in Table 5. Notably, the use of glucocorticoids and gabapentinoids was significantly more frequent among individuals with chronic chikungunya disease (CCD) compared to controls, particularly in the polyarthritis and oligoarthritis subgroups. While analgesic and NSAIDs use was common across all participants, the prescription of neuromodulatory agents was more prominent in phenotypes characterized by inflammation.

**Figure 2:**
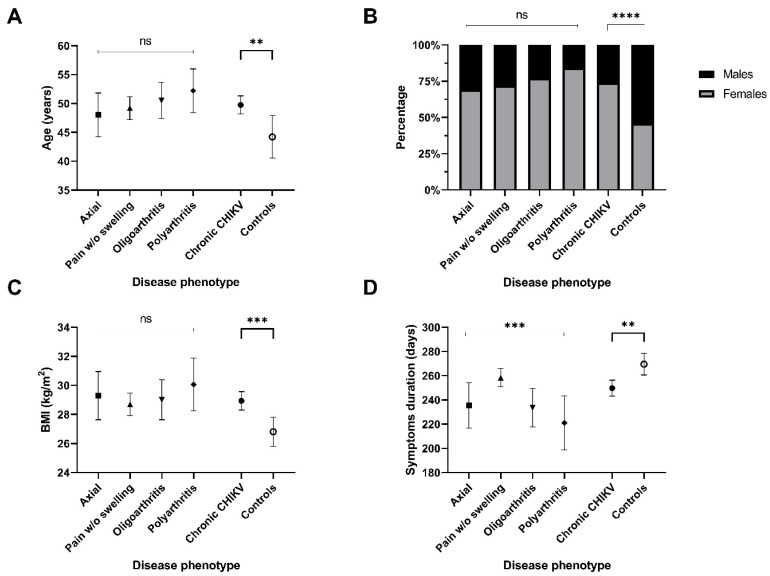
Clinical and demographic characteristics of patients with chronic chikungunya disease (CCD) compared to controls, and across CCD phenotypes. (**A**) Age boxplots in different disease phenotypes; (**B**) Sex proportions in disease phenotypes; (**C**) Body Mass Index boxplots in disease phenotypes; (**D**) Symptoms duration boxplots in disease phenotypes. BMI: Body Mass Index. ** *p* < 0.01; *** *p* < 0.001; **** *p* < 0.0001.

**Figure 3:**
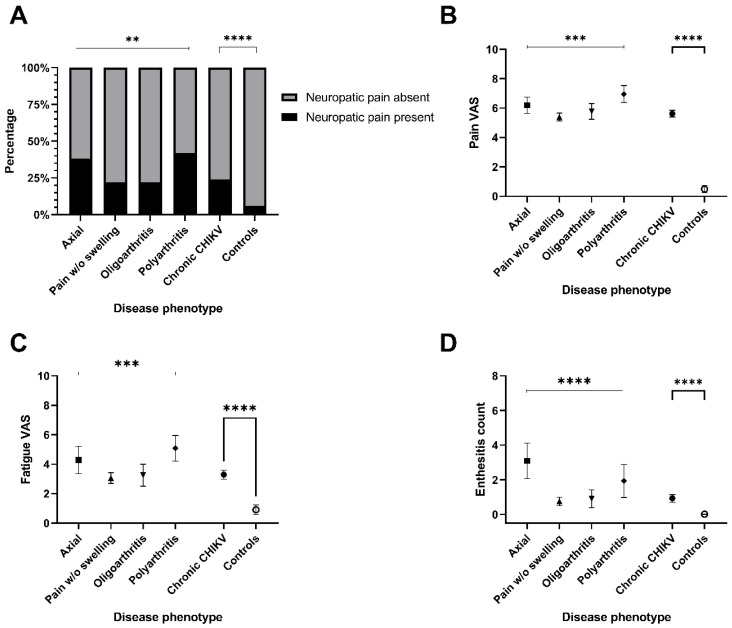
Comparisons in pain characteristics and fatigue between CCD and controls and across CCD phenotypes. (**A**) Proportion of neuropatic pain occurrence in disease phenotypes; (**B**) Pain (VAS) boxplots for disease phenotypes; (**C**) Fatigue VAS boxplots for disease phenotypes; (**D**) Enthesitis count boxplots for disease phenotypes. BMI: Body Mass Index ** *p*-value < 0.01; *** *p*-value < 0.001; **** *p*-value < 0.0001.

**Figure 4:**
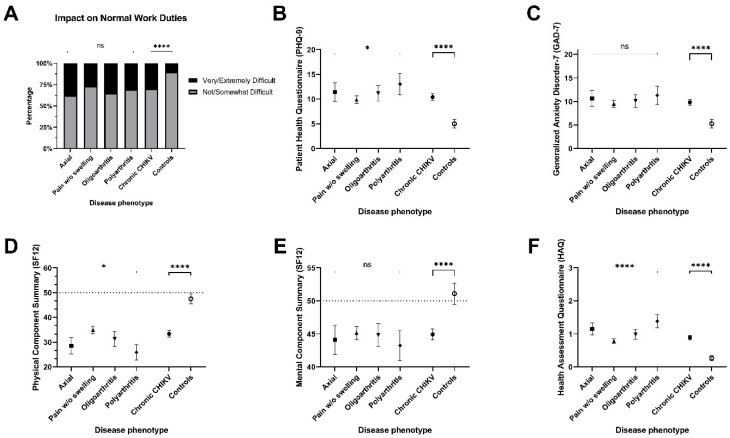
Functional status, disease burden, and mental health outcomes across patients with chronic chikungunya disease (CCD), controls, and CCD phenotypes. (**A**) Proportion of patients with assessed normal work duties as Not/Somewahat difficult vs. Very/Extremely dificult for disease phenotypes; (**B**) PHQ-9 boxplots for disease phenotypes; (**C**) GAD-7 boxplots for disease phenotypes; (**D**) Physical Component of SF12 boxplots for disease phenotypes; (**E**) Mental Component of SF12 boxplots for disease phenotypes; (**F**) HAQ boxplots for disease phenotypes. * *p* < 0.05 ; **** *p* < 0.0001.

Univariate comparisons between CCD phenotypes are shown in Figure 2, Figure 3 and Figure 4. No statistically significant differences in age, sex, or BMI were observed across phenotypes (Figure 2, panels A–C). However, symptom duration was significantly longer in the Pain without Swelling group compared to the Oligoarthritis and Polyarthritis subgroups (panel D).

Neuropathic pain was more frequent in Axial and Polyarthritis phenotypes (Figure 3, panel A), which also showed higher VAS scores for pain and fatigue (panels B and C). Conversely, the Pain without Swelling phenotype had the lowest scores for both. Enthesitis counts were higher in the Axial group (panel D).

Regarding broader impact, work disability was similarly frequent across CCD phenotypes (Figure 4, panel A). Depressive symptoms (PHQ-9) were more severe in the Polyarthritis group than in the Pain without Swelling group (panel B), while anxiety (GAD-7) and mental health scores (SF-12) did not differ significantly among the phenotypes (panels C and E). In contrast, physical health (SF-12) and functional disability (HAQ) were more impaired in Axial and Polyarthritis phenotypes (panels D and F).

In multinomial logistic regression analyses, distinct clinical profiles were associated with each CCD phenotype. In regression analyses, distinct clinical profiles were associated with each CCD phenotype. The Pain without Swelling phenotype was associated with longer symptom duration (OR 1.03 per week, 95% CI 1.01–1.06), lower physician-assessed disease activity (OR 0.78, 95% CI 0.70–0.86), and lower functional impairment measured by HAQ (OR 0.63, 95% CI 0.44–0.90). Oligoarthritis was associated with shorter symptom duration (OR 0.97 per week, 95% CI 0.95–1.00) and higher patient-reported global health score (OR 1.16, 95% CI 1.05–1.27). The Axial phenotype was associated with enthesitis (OR 2.73, 95% CI 1.17–6.34), higher enthesitis count (OR 1.20, 95% CI 1.05–1.36), and higher physician-assessed disease activity (OR 1.33, 95% CI 1.16–1.52). Polyarthritis was associated with higher physician-assessed disease activity (OR 1.55, 95% CI 1.33–1.82) and greater functional impairment measured by HAQ (OR 2.16, 95% CI 1.23–3.81).

## 4. Discussion

This study provides a comprehensive assessment of chronic chikungunya-associated disease (CCD), highlighting its phenotypic heterogeneity and the substantial burden on patients’ physical and mental health.

In line with many previous studies, our cohort demonstrated a predominance in CCD group of female patients, with older age and higher mean BMI compared to controls [10,14,15,16,20,22,23,28]. In addition, CCD was associated with a shorter interval since infection onset compared with controls. Although the cross-sectional design precludes temporal inferences, this finding may reflect a lower frequency of persistent symptoms among individuals evaluated later after acute infection, consistent with observations from previous longitudinal studies and meta-analyses of chikungunya outcomes [37].

Overall, patients with CCD exhibited a substantially higher burden of pain, fatigue, functional impairment, and objective inflammatory manifestations, including peripheral joint involvement, enthesitis, axial inflammatory pain, and neuropathic features, compared with controls. Despite this consistent clinical impact, systemic inflammatory activity assessed by C-reactive protein did not differ between groups. Previous studies have reported heterogeneous findings regarding systemic inflammatory biomarkers in chronic chikungunya disease: while some cohorts described normal or non-discriminatory CRP and ESR levels even in clinically active disease [38], others suggested associations with elevated CRP or ferritin in specific phases of infection or chronic arthralgia, albeit with limited consistency and modest prognostic performance [39,40]. The absence of significant CRP differences in our cohort may also reflect the heterogeneous timing of evaluation after acute infection and the limited sensitivity of isolated systemic biomarkers to capture localized or fluctuating inflammatory manifestations in CCD. Altogether, these data support the notion that clinical phenotyping may provide a more clinically meaningful framework than isolated laboratory markers in CCD.

Our phenotype-based approach highlights the marked clinical heterogeneity of chronic chikungunya disease, moving beyond a binary CCD-versus-control comparison. Rather than representing a single continuum of severity, the identified phenotypes appear to capture partially overlapping but biologically and clinically distinct patterns of disease expression. The Pain without Swelling phenotype, identified in the majority of CCD cases, was characterized by longer symptom duration and lower inflammatory and functional burden, suggesting a predominantly non-inflammatory or post-inflammatory pain state. In contrast, the Oligoarthritis phenotype clustered with shorter disease duration and better patient-reported global health, potentially reflecting a more limited or transitional inflammatory presentation. Differences in symptom duration across phenotypes may indicate clinically relevant heterogeneity in disease expression, but longitudinal studies are needed to determine whether these patterns represent stable phenotypes or transitions over time.

Axial and Polyarthritis phenotypes emerged as clinically closer entities, both associated with higher physician-assessed disease activity and greater overall disease impact, yet differing in their dominant drivers of disability. While the axial phenotype was strongly linked to enthesitis burden, the polyarthritis phenotype showed the most pronounced functional impairment, reinforcing the concept that inflammatory load and functional consequences may dissociate across CCD subtypes. Importantly, overlap between axial and peripheral manifestations suggests that these phenotypes may represent partially interconnected clinical dimensions rather than entirely isolated disease patterns, with potential implications for longitudinal disease evolution and treatment response.

The phenotypic patterns identified in our cohort are broadly consistent with previous attempts to classify chronic chikungunya disease, which have described three to five partially overlapping clinical phenotypes, ranging from isolated or mild arthralgia to more persistent and inflammatory joint involvement [13,41,42,43]. Across different classification systems, disease severity has been primarily driven by symptom persistence, extent of joint involvement, and functional impact, rather than by uniform laboratory abnormalities. Despite methodological heterogeneity and variability in phenotype definitions, these studies consistently support the concept that CCD is not a homogeneous condition but encompasses distinct clinical presentations with variable burden and prognosis.

Collectively, these phenotypes suggest that multiple pain mechanisms may coexist and contribute to the diverse clinical expressions of CCD, ranging from predominantly inflammatory manifestations to mixed inflammatory–neuropathic or post-inflammatory pain states. These patterns are compatible with mechanisms previously proposed in chronic chikungunya disease, including persistent or dysregulated immune activation following acute infection. Experimental, transcriptomic, and genetic studies have suggested roles for sustained inflammatory signaling, incomplete viral clearance, and host susceptibility factors in disease chronification [26,44,45,46,47]. However, the present study was not designed to explore pathophysiological mechanisms, and no mechanistic or causal inferences can be drawn from these clinical observations.

This study should be interpreted in light of certain limitations. As a cross-sectional analysis, it precludes causal inferences and limits conclusions regarding the temporal stability, evolution, or prognostic significance of the identified CCD phenotypes. Previous treatments were not controlled and may have transiently modified clinical manifestations, particularly objective inflammatory findings, potentially influencing phenotype allocation and potentially influencing phenotype allocation at the time of assessment. In addition, biomarkers were assessed at a single time point and may not fully capture the dynamic nature of inflammatory processes in chronic chikungunya disease. The study is also subject to selection bias, as inclusion depended on valid contact information and willingness to participate, potentially favoring individuals with greater healthcare engagement or persistent symptoms. Finally, information regarding the acute phase of disease was subject to patient recall bias, potentially limiting generalizability.

Notwithstanding these limitations, this study has several important strengths. It represents one of the largest clinically characterized CCD cohorts to date, with systematic rheumatologic evaluation, standardized and validated patient-reported outcomes, and the inclusion of a control group. Importantly, the phenotype-based analytical framework moves beyond traditional binary classifications and provides a clinically meaningful structure to interpret the heterogeneity of chronic chikungunya disease. This approach offers a robust foundation for future longitudinal studies aimed at validating phenotype stability, elucidating underlying mechanisms, and informing phenotype-oriented therapeutic strategies.

Taken together, these findings support the clinical relevance of phenotypic stratification in CCD, with implications for prognosis, therapeutic decision-making, and interpretation of treatment patterns. This framework provides a structured basis for understanding why patients with similar exposure to CHIKV may exhibit distinct clinical presentations, and it supports a more individualized approach to evaluation and management in chronic chikungunya disease.

## 5. Conclusions

In this Brazilian observational cohort, chronic chikungunya disease was shown to be a clinically heterogeneous condition with distinct and partially overlapping phenotypic presentations. Phenotypic stratification identified meaningful differences in symptom burden, functional impairment, and patterns of inflammatory and non-inflammatory pain, which were not captured by isolated laboratory markers. These findings reinforce the value of a phenotype-based clinical approach to CCD and support its potential relevance for prognosis, individualized management, and the design of future longitudinal and interventional studies.

## Figures and Tables

**Table 1:** Interpretation of questionnaires HAD, PHQ-9, SF12, and GAD-7.

HAQ	PHQ-9	SF-12	GAD-7
0–0.5: Almost normal function 0.5–1.0: Mild disability 1.0–2.0: Moderate disability 2.0–3.0: Severe disability	0–4: No depression 5–9: Mild depression 10–14: Moderate depression 15–19: Moderate to severe depression 20–27: Severe depression	>50: Above average 40–50: Mild impairment <40: Significant impairment	0–4: Minimal or absent anxiety 5–9: Mild anxiety 10–14: Moderate anxiety 15–21: Severe anxiety

HAQ: Health Assessment Questionnaire; PHQ-9: Patient Health Questionnaire-9; SF-12: 12-Item Short-Form Health Survey; GAD-7: Generalized Anxiety Disorder-7.

**Table 2:** Comparison of demographics and clinical characteristics of CCD and Control groups.

		Control Group *n* = 158	CCD Group *n* = 426	*p* Value
Age (mean ± SD), years		44.3 ± 23.4	49.8 ± 16.4	*p* = 0.001
Male sex, *n* (%)		53.8	25.8	*p* < 0.001
Race, *n* (%)				*p* = 0.409
	White	23.2	28.8	
	Asian	0.6	1.2	
	Indigenous	0.6	0.2	
	Brown	58.7	50.7	
	Black	16.8	19.1	
BMI (mean ± SD), kg/m^2^		26.8 ± 6.4	28.9 ± 6.6	*p* < 0.001
Time since infection onset (mean ± SD), days		268.7 ± 56.7	249.8 ± 69.6	*p* = 0.001
Hospitalization during acute phase, *n* (%)		8.2	4.2	*p* = 0.401

CCD: Chronic Chikungunya Disease; BMI: Body Mass Index. Race was self-reported according to IBGE classification.

**Table 3:** Standardized questionnaires assessing functionality, quality of life, and mental health in CCD and control groups.

Questionnaires	Controls	CCD	*p* Value
HAQ (mean ± SD)	0.3 ± 0.5	0.9 ± 0.7	*p* < 0.001
SF-12-PCS (mean ± SD)	47.5 ± 13.0	33.4 ± 13.9	*p* < 0.001
SF-12-MCS (mean ± SD)	51.1 ± 10.2	44.9 ± 8.7	*p* < 0.001
PHQ-9 (mean ± SD)	5.0 ± 5.6	10.4 ± 6.9	*p* < 0.001
GAD-7 total (mean ± SD)	5.3 ± 5.8	9.8 ± 6.4	*p* < 0.001

CCD: Chronic Chikungunya Disease; HAQ: Health Assessment Questionnaire; PHQ-9: Patient Health Questionnaire-9; SF-12-PCS: 12-Item Short-Form Health Survey Physical Component Score; SF12-MCS: 12-Item Short-Form Health Survey Mental Component Score; GAD-7: Generalized Anxiety Disorder-7.

**Table 4:** Comparison of the physical examination characteristics and the inflammatory biomarker values of CCD and Control groups.

Variables	Controls *n* = 158	CCD *n* = 426	*p* Value
Neuropathic pain *n* (%)	9 (5.7)	103 (24.2)	*p* < 0.001
VAS pain (mean ± SD)	0.5 ± 1.6	5.6 ± 2.4	*p* < 0.001
VAS fatigue (mean ± SD)	0.9 ± 2.1	3.3 ± 3.2	*p* < 0.001
VAS global activity by patient (mean ± SD)	0.7 ± 1.8	5.1 ± 2.5	*p* < 0.001
VAS global activity by physician (mean ± SD)	0.2 ± 0.7	2.8 ± 2.3	*p* < 0.001
TJC-68 (mean ± SD)	0 ± 0	5.7 ± 8.7	*p* < 0.001
SJC-66 (mean ± SD)	0 ± 0	1.1 ± 2.9	*p* < 0.001
Enthesitis presence, *n* (%)	2 (1.3)	104 (24.4)	*p* < 0.001
enthesitis count (mean ± SD)	0.0 ± 0.1	0.9 ± 2.3	*p* < 0.001
CTS presence, *n* (%)	3 (1.9)	46 (10.8)	*p* < 0.001
Inflammatory Axial Pain presence, *n* (%)	0 (0)	52 (12.2)	*p* < 0.001
CRP (mean ± SD)	0.4 ± 0.7	0.6 ± 1.3	*p* = 0.281

CCD: Chronic Chikungunya Disease; Neuropathic pain was considered when DN-4 questionnaire ≥ 4; VAS: Visual Analog Scale. TJC-68: Tender Joint Count in 68 joints; SJC-66: Swollen Joint Count in 66 joints; CTS: Carpal Tunnel Syndrome; CRP: C-reactive Protein.

**Table 5:** Medications in use prior to visit 1 by group.

	Glucocorticoids (%)	Analgesics/NSAIDs (%)	Gabapentinoids (%)	Other (%)
Control group	20.4	90.1	1.8	1.5
CCD (total)	39.1 ***	96.7 **	7.3 **	6.0 **
Pain w/o swelling	37.8 ***^/‡^	93.9 ^‡^	0.9 ^‡^	2.4 ^‡^
Axial	42.8 *	97.9 *	6.0 *	6.0 *
Oligoarthritis	33.3 ^§^	97.4	15.3 ***	8.9 **
Polyarthritis	55.5 ***	97.7 *	8.8 **	11.1 **

CCD: Chronic Chikungunya Disease; NSAIDs: nonsteroidal anti-inflammatory drugs. Values are presented as percentages. Comparisons between each CCD subgroup and the control group were performed using Pearson’s chi-square test or Fisher’s exact test, as appropriate. * *p* < 0.05 vs. control group; ** *p* < 0.01 vs. control group; *** *p* < 0.001 vs. control group; ^‡^ *p* < 0.05 for comparisons between the Pain without Swelling phenotype and inflammatory phenotypes; ^§^ *p* < 0.05 for comparisons between the Oligoarthritis and Polyarthritis phenotypes. No formal correction for multiple comparisons was applied due to the exploratory nature of the analyses.

## Data Availability

Data used in this study is not currently available. The authors may provide the data after formal request.

## Supplementary Materials

The following supporting information can be downloaded at: https://www.mdpi.com/article/10.3390/tropicalmed11050140/s1, Table S1: STROBE checklist.

## Abbreviations

The following abbreviations are used in this manuscript:

BMI	Body Mass Index
CAAE	Certificado de Apresentação de Apreciação Ética
CCD	Chronic Chikungunya Disease
CHIKF	Chikungunya Fever
CHIKV	Chikungunya Virus
CI	Confidence Interval
CRP	C-reactive Protein
CTS	Carpal Tunnel Syndrome
DN4	Douleur Neuropathique en 4 questions
ESR	Erythrocyte Sedimentation Rate
GAD-7	Generalized Anxiety Disorder-7
HAQ	Health Assessment Questionnaire
IBGE	Instituto Brasileiro de Geografia e Estatística
IgM	Immunoglobulin M
IL	Interleukin
INCT	Instituto Nacional de Ciência, Tecnologia e Inovação
MASES	Maastricht Ankylosing Spondylitis Enthesitis Score
NSAIDs	Non-Steroidal Anti-Inflammatory Drugs
OR	Odds Ratio
PCS	Physical Component Summary
PHQ-9	Patient Health Questionnaire-9
RT-PCR	Reverse Transcription Polymerase Chain Reaction
SD	Standard Deviation
SF-12	12-Item Short-Form Health Survey
SJC-66	Swollen Joint Count (66 joints)
SPARCC	Spondyloarthritis Research Consortium of Canada
STROBE	Strengthening the Reporting of Observational Studies in Epidemiology
TJC-68	Tender Joint Count (68 joints)
TNF	Tumor Necrosis Factor
VAS	Visual Analog Scale
