# Supplementary material for: Unveiling Clinical Phenotypes in Chronic Chikungunya Disease: Insights from a Brazilian Observational Study
Source: Trop Med Infect Dis. 2026 May 19;11(5):140. doi: 10.3390/tropicalmed11050140 (PMC13211594; doi:10.3390/tropicalmed11050140)
Supplement: Supplementary file 1 [file tropicalmed-11-00140-s001.zip › STROBE checklist (1).pdf]

STROBE Item	Recommendation	Reported on Page(s)
**Title and abstract**		
1(a)	Indicate the study's design with a commonly used term in the title or abstract	Title page / Abstract
1(b)	Provide in the abstract an informative and balanced summary of what was done and found	Abstract
**Introduction**		
2	Explain the scientific background and rationale for the investigation	Introduction
3	State specific objectives, including prespecified hypotheses	Introduction (final paragraph)
**Methods**		
4	Present key elements of study design early in the paper	Methods
5	Describe the setting, locations, and relevant dates, including recruitment and follow-up periods	Methods – Study design and participants
6(a)	Give eligibility criteria and sources/methods of participant selection	Methods – Participants
7	Clearly define outcomes, exposures, predictors, potential confounders, and diagnostic criteria	Methods – Clinical assessment and phenotype definitions
8*	For each variable of interest, give sources of data and details of assessment methods	Methods – Clinical and laboratory assessment
9	Describe efforts to address potential sources of bias	Methods / Discussion – Limitations
10	Explain how study size was determined	Methods
11	Explain how quantitative variables were handled in the analyses	Statistical analysis
12(a)	Describe all statistical methods, including those used to control for confounding	Statistical analysis
12(b)	Describe methods used to examine subgroups and interactions	Statistical analysis

12(c)	Explain how missing data were addressed	Statistical analysis
12(d)	If applicable, explain how loss to follow-up was addressed	Not applicable
12(e)	Describe any sensitivity analyses	Not applicable
**Results**		
13(a)	Report numbers of individuals at each stage of the study	Results
13(b)	Give reasons for non-participation at each stage	Results / Flowchart (if applicable)
13(c)	Consider use of a flow diagram	Figure 1
14(a)	Give characteristics of study participants	Table 1
14(b)	Indicate number of participants with missing data for each variable	Results / Tables
15	Report numbers of outcome events or summary measures	Results
16(a)	Give unadjusted and adjusted estimates with precision measures	Tables 3–5 / Results
16(b)	Report category boundaries when continuous variables were categorized	Methods / Tables
16(c)	If relevant, consider translating estimates into absolute risk	Not applicable
17	Report other analyses done, including subgroup analyses	Results
**Discussion**		
18	Summarize key results with reference to study objectives	Discussion
19	Discuss limitations of the study, considering potential bias or imprecision	Discussion – Limitations
20	Give a cautious overall interpretation of results	Discussion
21	Discuss generalizability of the results	Discussion
**Other information**		
22	Give the source of funding and role of funders	Funding / Acknowledgments
